# How much of the current serious arbovirus epidemic in Brazil is dengue and how much is chikungunya?

**DOI:** 10.1016/j.lana.2024.100753

**Published:** 2024-04-30

**Authors:** André Ricardo Ribas Freitas, Alessandro Aldrin Pinheiro Chagas, André Machado Siqueira, Luciano Pamplona de Góes Cavalcanti

**Affiliations:** a Faculdade São Leopoldo Mandic, Campinas, São Paulo, Brazil; b Conselho Nacional de Secretarias Municipais de Saúde – CONASEMS, Brazil; c Fundação Oswaldo Cruz, Instituto Nacional de Infectologia–INI, Rio de Janeiro, Brazil; d Universidade Federal do Ceará and Centro Universitário Christus, Fortaleza, Ceará, Brazil

Dengue remains a serious public health problem and until 2013, it was the only arbovirus transmitted by urban species of *Aedes* mosquitoes in the Western Hemisphere. Justifying the adoption of clinical and epidemiological criteria for reporting probable dengue cases, to overcome insufficient laboratory capacity and to increase sensitivity during large epidemic, probable cases are those reported as suspected dengue, regardless of laboratory.^1^ Chikungunya was introduced to the Americas at the end of 2023, with major epidemics occurring in 2014 and 2015, and high mortality rates documented in several locations,^2^ in others identified only by excess deaths.^3^ In Brazil, the spread of chikungunya is not uniform, leading to isolated epidemics each year.^4^

Unlike dengue, a case of chikungunya is only reported with more rigorous clinical suspicion and confirmation requires a positive laboratory test or very suggestive clinical suspicion linked to other laboratory-confirmed cases. There is considerable overlap in clinical symptoms between the two arboviruses^5^ and chikungunya is not well known by most healthcare professionals, leading to a bias in clinical suspicion, contributing to many cases of chikungunya being reported as dengue. Despite the introduction of RT-qPCR Multiplex tests–which identify dengue, Zika and chikungunya simultaneously–in official laboratories, during epidemics, the majority of patients do not undergo confirmatory tests and, therefore, end up being considered dengue fever.^1^ This reinforces the false idea that chikungunya continues to be a rare disease in Brazil.

Data from the state of Minas Gerais (MG) can be used to illustrate this point because of the excellent surveillance structure that systematically performs Multiplex RT-qPCR for etiological diagnosis of a large proportion of patients, regardless of the initial clinical suspicion. MG is also of the largest states in Brazil and concentrated 25% of the cases that occurred in 2023. To illustrate, we present in Table 1 the official data for the year 2023, for the State of Minas Gerais (MG) and its capital, Belo Horizonte (BH). In 2023, the absolute number of positive tests for chikungunya was 3.8 times higher than for dengue in the capital (3461 and 908 cases respectively) and 1.4 times higher in the state (36,668 and 26,885 cases respectively). The positivity of tests for chikungunya was 2.1–5.1 times higher than for dengue in the capital (BH) and in the state (MG) both in direct methods (RT-qPCR Multiplex) and indirect methods (enzyme immunoassay IgM). Paradoxically, the “official” number of “probable dengue” cases was 2.4 times greater than the number of chikungunya cases in BH (14,050 and 5962 respectively) and 5 times greater in MG (413,307 and 83,330 respectively). Projecting test positivity for the set of suspected arbovirus cases, we estimate approximately the most real number of chikungunya and dengue cases in BH and MG.

**Table 1:** Number of officially reported clinical cases, diagnostic tests performed in official laboratories and estimated clinical cases (State of Minas Gerais and Belo Horizonte, Brazil, 2023).

	Officially reported clinical cases		Multiplex RT-qPCR			Enzyme immunoassay (IgM) tests			Total positive tests (RT-qPCR and IgM)	Overall test positivity [(c) + (e)]/[(b) + (d)]	Rough estimate of real clinical cases^a^
	Suspects^c^ (a)	Probable^c^	Performed^d^ (b)	Positives^d^ (c)	Test positivity (c)/(b)	Performed^d^ (d)	Positives^d^ (e)	Test positivity (e)/(d)			
Belo Horizonte (BH)											
Dengue	53,405	14,050	8200	520	6.3%	3464	388	11.2%	908	7.8%	4769
Chikungunya	7861	5962	8198	1989	24.3%	2553	1472	57.7%	3461	32.2%	19,723
Arbovirus^b^ (total)	61,266	20,012							4932		24,492
Minas Gerais (MG)											
Dengue	699,559	413,307	83,206	9007	10.8%	65,447	17,878	27.3%	26,885	18.1%	149,868
Chikungunya	129,095	83,330	83,242	24,324	29.2%	21,808	12,344	56.6%	36,668	34.9%	289,244
Arbovirus^b^ (total)	828,654	496,637							63,553		439,112

a The estimate of real clinical cases was calculated by multiplying the number of suspected arboviruses by the overall positivity of laboratory tests.

b There may be patients who have been notified twice, for dengue and chikungunya.

c Sources: http://tabnet.datasus.gov.br (accessed on 03/19/2024).

d Sources: https://www.saude.mg.gov.br/aedes/painelvigilancialaboratorial (accessed on 03/19/2024).

Despite the initial similarity, chikungunya has been shown to be capable of inflicting a high mortality rate. The most common serious complication of dengue is hypovolemic shock. The main serious complications of chikungunya are cardiogenic shock, septic shock and encephalitis. One therapeutic approach for severe dengue includes high-volume hydration, which can be fatal for a patient with severe chikungunya. In addition to the harm to patient care, underestimation of chikungunya cases can divert investment priorities into research for treatment and immunization against this disease. We ask whether this same underestimation of chikungunya cases with the simultaneous inflation of dengue cases is not happening in other regions of Brazil and South America, which is experiencing one of the worst “dengue” epidemics in history.

## Contributors

ARRF, conceptualisation, data curation, formal analysis, writing—original draft; AAPC, writing—review; AMS, writing—review; LPGC, conceptualisation, writing—original draft. All the authors approved the final submission of the study and accept responsibility for the decision to submit for publication.

## Declaration of interests

The author or authors declare that they have no conflict of interest.
